# Surface-protein interactions on different stainless steel grades: effects of protein adsorption, surface changes and metal release

**DOI:** 10.1007/s10856-013-4859-8

**Published:** 2013-02-02

**Authors:** Y. Hedberg, X. Wang, J. Hedberg, M. Lundin, E. Blomberg, I. Odnevall Wallinder

**Affiliations:** 1 Division of Surface and Corrosion Science, Department of Chemistry, School of Chemical Science and Engineering, KTH Royal Institute of Technology, Drottning Kristinas väg 51, 100 44 Stockholm, Sweden; 2 YKI, Institute for Surface Chemistry, P.O. Box 5607, 114 86 Stockholm, Sweden

## Abstract

Implantation using stainless steels (SS) is an example where an understanding of protein-induced metal release from SS is important when assessing potential toxicological risks. Here, the protein-induced metal release was investigated for austenitic (AISI 304, 310, and 316L), ferritic (AISI 430), and duplex (AISI 2205) grades in a phosphate buffered saline (PBS, pH 7.4) solution containing either bovine serum albumin (BSA) or lysozyme (LSZ). The results show that both BSA and LSZ induce a significant enrichment of chromium in the surface oxide of all stainless steel grades. Both proteins induced an enhanced extent of released iron, chromium, nickel and manganese, very significant in the case of BSA (up to 40-fold increase), whereas both proteins reduced the corrosion resistance of SS, with the reverse situation for iron metal (reduced corrosion rates and reduced metal release in the presence of proteins). A full monolayer coverage is necessary to induce the effects observed.

**Electronic supplementary material:**

The online version of this article (doi:10.1007/s10856-013-4859-8) contains supplementary material, which is available to authorized users.

## Introduction

Protein-induced metal release from stainless steels is of high importance as these alloys are commonly used as implant materials [1, 2], and in food processing applications [3]. Stainless steels are iron-based alloys with significant amounts (>approx. 11 wt%) of chromium, more than 8 wt% nickel in the case of austenitic stainless steels, and contain many minor elements such as molybdenum and manganese [4]. A high corrosion resistance is related to the formation of a chromium(III)-rich passive surface oxide [4, 5]. The characteristics of this passive oxide are very similar to the surface oxide formed on chromium metal from which minor amounts of chromium are released when exposed to biological media [6–8]. Previous findings for inert-gas atomized stainless steel powders and massive sheets have proven chromium to be released as chromium(III), not chromium(VI), upon exposure in synthetic biological fluids [8–11].

Surface interactions and adsorption of proteins vary strongly due to their heterogeneous nature. For stainless steels, both physisorption and irreversible chemisorption of proteins may be possible [12–14]. Surface complexation between serum proteins and the surface metal hydroxide has been suggested as the main reason for metal released from stainless steel (316) powder (55 μm) at both in vitro and in vivo conditions [15]. Monolayer adsorption onto hydrophilic surfaces is often reported for bovine serum albumin (BSA) or human serum albumin (HSA) [16–18], while the smaller protein lysozyme (LSZ) may adsorb in several layers [16, 19]. Both LSZ and BSA (and HSA) have been reported to adsorb on stainless steel (316) exposed to PBS at pH 7.4 (and at pH 4.0), albumin as a monolayer and the significantly smaller protein LSZ in several layers [20]. BSA is net negatively charged (iep 4.7–5.2) [21] and LSZ net positively charged (iep 11) at pH 7.4 [22]. The normal albumin concentration in human plasma is 42 ± 3.5 g/L [21], while the human LSZ concentration in serum is 7–13 mg/L and about 1.2 g/L in tear fluid [23].

Since the release of metals from metals and alloys often is the result of several pooled processes including chemical, electrochemical, and physical processes, corrosion and metal release are not the same, at least not at ambient conditions, i.e. no applied potential [24, 25]. Contradictory findings are reported in the literature related to the release of metals from metals and alloys in biological fluids containing proteins [5, 26–31] or complexing agents [5, 26, 32, 33]. Both increased metal release [5, 15, 26, 28, 31, 33] and corrosion [27, 29, 32] and decreased metal release [28] and corrosion [27, 30] of alloys, such as stainless steels [5, 15, 26, 28, 30–33], carbon steel [27] and pure metals [26] have been reported. Two different important mechanisms have been proposed to explain the effect of surface-protein interactions on the metal release process: (i) enhanced metal release due to surface-induced complexation [15, 26, 32] and (ii) inhibition of metal release due to the adsorption of rigid protein layers [27, 28]. So far, limited mechanistic metal release studies exist that correlate protein adsorption with changes in surface oxide composition, microstructure and corrosion properties.

The objective of this study is to fill this knowledge gap through an in-depth investigation of the correlation between protein adsorption, which is elucidated using BSA and LSZ due to their large differences in adsorption properties, size and charge, and release of metals (iron, chromium, nickel, and manganese) from stainless steel grades of different microstructure exposed in PBS (pH 7.4) for periods up to one week. These include austenitic stainless steels (AISI 304, 310, and 316L), nickel-free ferritic stainless steel (AISI 430), duplex stainless steel (AISI 2205) and iron metal.

## Materials and methods

### Materials

The study comprises stainless steel grades of different microstructure, supplied from ThyssenKrupp, Germany, and iron metal for comparative reasons. Grade, microstructure and composition, based on supplier information, are given in Table 1.

**Table 1:** Grade, microstructure, and bulk composition (wt%) of investigated stainless steel alloys and iron metal, based on supplier information

Structure	Grade(AISI)	Cr	Fe	Mn	Ni	Mo	C	P	Si	S
Ferritic	430	16.0	Balance	0.5	0.22	0.06	0.04	0.02	0.3	0.002
Austenitic	304	18.1	Balance	1.1	9.0	0.3	0.05	0.03	0.3	0.002
Austenitic	310	24.2	Balance	0.9	19.1	0.2	0.06	0.02	0.4	0.001
Austenitic	316L	16.6	Balance	1.0	10.6	2.1	0.03	0.02	0.4	0.001
Duplex	2205	22.5	Balance	1.7	5.6	3.1	0.02	0.03	0.3	0.001
	Iron	–	Balance	0.2	–	–	0.06	0.01	0.01	0.01

### Metal release test procedure and analysis

Double sided coupons of each material (thickness 1 mm) were prepared with an approximate size of 1.5 × 1.5 cm^2^ (approximately 4–6 cm^2^ in total, including both sides). Prior to exposure, all surfaces were abraded using 1200 grit SiC paper, ultrasonically cleaned in acetone and isopropyl alcohol for 7 min, respectively, and dried with cold nitrogen gas. This preparation step was followed by ageing for 24 h in a desiccator (at room temperature) prior to the immersion experiments. The coupons were positioned vertically (i.e. the entire surface area exposed) in polyethylene vessels. The surface area to solution volume ratio was kept constant at 1 cm^−1^ (1 cm^2^/1 mL). Triplicate samples and one blank sample (solution without any metal coupon) were exposed in parallel for each material, time period, and solution, respectively. Exposures were conducted in a Stuart platform-rocker incubator (37 ± 0.5 °C, 25 cycles/min of bilinear shaking) for time periods up to 1 week (168 h): 2, 4, 8, 24, and 168 h for grades 304, 316L, and 430, and 2 and 168 h for grades 310 and 2205. Three main solutions were used: PBS buffer (8.77 g/L NaCl, 1.28 g/L Na_2_HPO_4_, 1.36 g/L KH_2_PO_4_, 370 μL/L 50 % NaOH, pH 7.2–7.4, Sigma-Aldrich) only, denoted PBS, PBS buffer with 10 g/L BSA (A7906, Sigma-Aldrich), denoted PBS + BSA, and PBS buffer with 2.2 g/L LSZ (L6876, Sigma-Aldrich), denoted PBS + LSZ. The molar concentration of BSA and LSZ was identical. The importance of BSA concentration (0.01, 0.1, 1, 10 (standard), and 100 g/L) was elucidated for 316L immersed for 168 h in PBS. The release of iron from iron metal was investigated in PBS and PBS + BSA after 24 h of exposure. All protein solutions were equilibrated for 1 h in order to ensure complete dissolution and freshly prepared or frozen prior to exposure in order to avoid protein denaturation.

All vessels and tools were acid-cleaned in 10 % HNO_3_ for at least 24 h, rinsed four times in ultrapure water (resistivity of 18.2 MΩcm), Millipore Sweden, and dried in ambient laboratory air. All chemicals used were of analytical grade (p.a.) or puriss p.a. grade (in the case of nitric acid used for sample acidification).

The solution pH was measured in all test solutions prior to and after exposure. A change of less than 0.1 pH was observed in all cases, with the exception of iron metal for which the pH increased with approx. 3 pH units in PBS (24 h) and with 0.5–1 pH unit in PBS + BSA (24 h). All samples were acidified (with 65 % nitric acid) to a pH less than 2 for accurate metal analysis.

Graphite furnace atomic absorption spectroscopy (GF-AAS) or flame atomic absorption spectroscopy (AAS), using a Perkin–Elmer AAnalyst 800 instrument, were used to determine the released metal concentration in μg/L or mg/L, respectively. Released concentrations of iron, chromium, nickel, and manganese were analyzed using standard operational procedures (Perkin–Elmer), with limits of detection, LOD, of 18 μg/L for iron, 0.98 μg/L for chromium, 10 μg/L for nickel, and 0.11 μg/L for manganese. The exception was nickel for which a higher atomization temperature (2,400 °C) was used. Calibration was done using a blank (ultrapure water) and at least three calibration standards. Quality control samples were analyzed consecutively, approximately every 10th sample.

Data presented represent the mean values of three replicate samples with the blank value for the given exposure subtracted. The reason for any higher blank concentrations of the protein solutions, compared with the PBS solution, was related to the natural metal content of the proteins [20], 0 and 5 μg Cr/L for BSA and LSZ, respectively, non-significant for Fe (analysed by flame-AAS), and <LOD for Ni and Mn (BSA and LSZ, respectively).

### Surface analysis

Surface compositional analysis was performed using X-ray photoelectron spectroscopy, XPS. Spectra were recorded using a Kratos AXIS UltraDLD X-ray photoelectron spectrometer (Kratos Analytical) using a monochromatic Al X-ray source (150 W) on areas approximately sized 700 × 300 μm^2^. Wide spectra were run to detect elements present in the outermost surface oxide (information depth of a few nanometers) at five different locations. High resolution spectra (20 eV pass energy) were acquired for the main bulk compositional elements Cr 2p, Fe 2p, Ni 2p, Mo 3d, O 1s of each test item including carbon (C 1s). The content of metals in the uppermost surface oxide was presented as the relative mass ratio of oxidized iron and chromium, Cr_ox_/(Cr_ox_ + Fe_ox_). Protein adsorption was described by the relative atomic ratio between N (N 1s) and the oxidized carbon C 1s peaks (C2 + C3) as described in Sect. 3.1.1. Typical high resolution spectra for Fe 2p (a), Cr 2p (b), C 1s (c), and N 1s (d) are shown in Fig. S1, supplementary material. Other experimental details are given elsewhere [8, 34].

### Electrochemical measurements

The electrochemical measurements were performed with a Solartron electrochemical interface 1286 using a standard, three-electrode setup, using a saturated Ag/AgCl electrode as reference and a platinum mesh as counter electrode. The linear polarization resistance (LPR) was measured by polarizing the working electrode 15 mV in both positive and negative directions versus the open circuit potential (OCP) at a scan speed of 0.1667 mV/s. The LPR measurement took place after 18 h immersion to ensure OCP stabilization, and at that time the OCP change was about 1 mV/h. Thus, the LPR measurements were performed under quasi-equilibrium conditions where the OCP changed at a substantial lower rate than the applied potential, which is required for accurate LPR results. The experimental data was analyzed using CorrView software and the polarization resistance was calculated from the I-E polarization curves. The obtained I-E curves were linear in the interval selected, i.e. 15 mV in positive and negative directions from OCP. OCP experiments for up to 24 h of immersion and LPR experiments after 18 h of immersion were conducted in PBS for the grade 304 and iron metal, in PBS + BSA for the grade 304 and iron metal, and in PBS + LSZ for the grade 304. As for the metal release measurements, the stainless steel sheets were allowed to form a reproducible surface oxide for 24 h prior to exposure by grinding, cleaning and ageing described in Sect. 2.2, in order to investigate a relevant and well-defined surface condition.

### Protein adsorption measurements by means of Quartz crystal microbalance (QCM)

Adsorption studies were conducted by means of quartz crystal microbalance with dissipation monitoring using a QCM-D E4 instrument from Biolin Scientific (Sweden). The layer thickness of the adsorbed protein film was estimated by using the Voigt model introduced for estimation of QCM-D data [35], already described in detail for this system in [20]. A baseline was established for 1 h in the PBS solution at pH 7.4 and protein adsorption was allowed to proceed for 1 h. All measurements and the cleaning of the substrates were conducted according to the procedure described in [20] using a constant flow of 200 μL/min over an evaporated stainless steel (grade AISI 316) surface, purchased from Biolin Scientific, Sweden (QSX 304) at 37 °C. The protein concentration of LSZ (Sigma-Aldrich, L6876) or BSA (Sigma-Aldrich, A3912) was set to 1 g/L.

### Zeta potential measurements

An electrokinetic analyzer (SurPASS, Anton Paar GmbH, Graz, Austria) was used to measure the zeta potential of the QCM stainless steel (316) surfaces, cleaned according to the procedure described in [20]. Detailed information on the instrumental technique and set-up can be found elsewhere [36, 37]. In brief, an adjustable-gap cell for circular discs with a diameter of 1.4 cm was used and the variable separation between the two stainless steel surfaces was set to approximately 120 μm. Flow was induced in the measurement cell by ramping the differential pressure from 0 to 300 mbar in both flow directions. The zeta potential was determined from the Smoluchowski equation by measuring the change in streaming current versus the applied differential pressure [37]. A 1 mM KCl solution was used as electrolyte and HCl (0.1 M) and NaOH (0.1 M) were used to adjust the pH in the range between 2 and 10.

### Statistical analysis

The significance of any differences between different sample sets was calculated by means of a Student’s *t* test (unpaired data with unequal variance). The lowest *P* value presented is 0.0001 and differences are referred to as significant for *P* < 0.05, with higher significance for lower *P* values.

## Results and discussion

### Protein-induced metal release from different stainless steel grades

#### Metal release and surface oxide composition

Previous findings by the authors have shown the presence of different proteins (BSA/HSA, LSZ, Mucin) in PBS solutions of both pH 7.4 and pH 4.0 [5, 20] to enhance the release of iron from stainless steel grade 316L, results in agreement with other literature findings [15, 26, 31]. A similar approach was adopted in this study to verify if other stainless steel grades of different microstructure and corrosion resistance, including ferritic (430), austenitic (304, 310, 316L) and duplex (2205), grades of increasing alloy content and generally improved corrosion properties, would show similar results. The same trend with enhanced released amounts of metals in the presence of proteins, as previously observed for some grades, was evident for all grades, independent of microstructure, Table 2. In general, all alloy constituents investigated (Fe, Cr, Ni, and Mn) were released to a significantly higher extent in the protein (BSA or LSZ) containing PBS solution compared to the non-protein containing solution of the same pH. BSA induced a significantly higher extent of released metals compared to LSZ, for all stainless steel grades investigated. The extent of metal release into PBS + BSA, compared to PBS, was enhanced by a factor of 2–39 for grade 430, 7–36 for grade 304, 3–15 for grade 316L, 2–36 for grade 310, and 0.5–9 for grade 2205, Table 2. The released metal quantity into PBS + LSZ, compared to PBS, was in contrast only significantly enhanced for some elements and stainless steel grades; by a factor of 0.4–3 for 430, 0.9–21 for 304, 0.2–2 for 316L, 0.7–4 for 310, and 0.5–2.4 for 2205, Table 2.

**Table 2:** Release of iron, chromium, nickel, and manganese (μg/cm^2^) from stainless steel grades 430, 304, 316L, 310, and 2205 into PBS, PBS + BSA, and PBS + LSZ, presented as average values and standard deviation between triplicate samples

Stainless steel grade	Released element	Exposure time period (h)	PBS	PBS + BSA	PBS + LSZ
430	Fe	2	0.035 ± 0.005	1.4 ± 0.02***	0.078 ± 0.003**
		168	0.42 ± 0.04	4.7 ± 0.2***	0.93 ± 0.1**
	Cr	2	0.00038 ± 0.0002	0.0078 ± 0.0008**	0.00019 ± 0.0002
		168	0.0020 ± 0.0004	0.030 ± 0.002***	0.0028 ± 0.0002
	Ni	2	<LOD	<LOD	<LOD
		168	<LOD	0.00031	<LOD
	Mn	2	0.0052 ± 0.0006	0.0088 ± 0.0004**	0.0021 ± 0.002
		168	0.0034 ± 0.0006	0.011 ± 0.002**	0.011 ± 0.002*
304	Fe	2	0.038 ± 0.005	0.90 ± 0.03***	0.082 ± 0.003***
		168	0.27 ± 0.02	1.9 ± 0.02***	0.79 ± 0.02***
	Cr	2	0.00060 ± 0.00004	0.0060 ± 0.0006**	0.00055 ± 0.00008
		168	0.0019 ± 0.0002	0.018 ± 0.005*	0.0060 ± 0.0007**
	Ni	2	0.00092 ± 0.0003	0.018 ± 0.002**	0.0013 ± 0.0003
		168	0.0012 ± 0.001	0.036 ± 0.002***	0.0042 ± 0.0006
	Mn	2	0.0083 ± 0.0006	0.022 ± 0.004*	0.013 ± 0.003
		168	0.0075 ± 0.0004	0.020 ± 0.0005***	0.015 ± 0.0009**
316L	Fe	2	0.045 ± 0.009	0.68 ± 0.2*	0.032 ± 0.003
		168	0.41 ± 0.03	2.3 ± 0.05***	0.41 ± 0.02
	Cr	2	0.00045 ± 0.0002	0.0057 ± 0.0009**	0.000080 ± 0.00004
		168	0.0012 ± 0.0002	0.015 ± 0.0009**	0.0024 ± 0.0006
	Ni	2	0.0021 ± 0.001	0.022 ± 0.02	0.0033 ± 0.009
		168	0.011 ± 0.001	0.10 ± 0.006***	0.010 ± 0.004
	Mn	2	0.0034 ± 0.0004	0.011 ± 0.004	0.0029 ± 0.002
		168	0.0096 ± 0.002	0.028 ± 0.002***	0.011 ± 0.001
310	Fe	2	0.032 ± 0.003	0.36 ± 0.10*	N/A
		168	0.20 ± 0.02	0.74 ± 0.03***	0.37 ± 0.02***
	Cr	2	0.00010 ± 0.0001	0.0037 ± 0.002	N/A
		168	0.0036 ± 0.0003	0.0091 ± 0.001*	0.0045 ± 0.0004*
	Ni	2	0.0052 ± 0.0003	0.016 ± 0.009	N/A
		168	0.026 ± 0.0006	0.089 ± 0.01**	0.10 ± 0.005**
	Mn	2	0.0028 ± 0.0001	0.013 ± 0.003*	N/A
		168	0.0055 ± 0.0009	0.011 ± 0.002*	0.0040 ± 0.0002
2205	Fe	2	0.021 ± 0.007	0.19 ± 0.04*	N/A
		168	0.26 ± 0.02	1.4 ± 0.05***	0.58 ± 0.04**
	Cr	2	0.00052 ± 0.0002	0.0038 ± 0.0006**	N/A
		168	0.0025 ± 0.0003	0.015 ± 0.0004***	0.0050 ± 0.0005*
	Ni	2	<LOD	0.038 ± 0.03	N/A
		168	0.0030 ± 0.003	0.023 ± 0.0006**	0.0014 ± 0.0002
	Mn	2	0.011 ± 0.001	0.0050 ± 0.0008**	N/A
		168	0.0076 ± 0.0004	0.023 ± 0.0005***	0.019 ± 0.001***

The difference to the corresponding PBS sample values, determined by a Student’s *t* test (unpaired data with unequal variance) is indicated by *asterisks*, * *P* < 0.05, ** *P* < 0.01 and *** *P* < 0.001

<*LOD* below limit of detection, *N/A* no data available

Even though the exposures in protein solutions resulted in a significantly enhanced release of individual alloy components from the different stainless steel grades, this release corresponds to only very small fractions of the total amount of metals in each alloy (<0.0023 %). Quantitative findings of the total amount of released metals (Fe + Cr + Ni + Mn) from each grade exposed in PBS with and without proteins for 168 h showed decreasing amounts according to following sequences:

PBS (μg/cm²): 316L^A^(0.43) ≈ 430^F^(0.43) > 304^A^(0.28) ≈ 2205^D^(0.27) ≈ 310^A^(0.24)
PBS + BSA (μg/cm^2^): 430^F^(4.7) > 316L^A^(2.4) > 304^A^(2.0) > 2205^D^(1.43) > 310^A^(0.85)
PBS + LSZ (μg/cm^2^): 430^F^(0.94) ≈ 304^A^(0.81) > 2205^D^(0.61) > 310^A^(0.48) > 316L^A^(0.44)

A, F and D denotes austenitic, ferritic and duplex grades, respectively. “≈” denotes non-significant (*P* > 0.05) differences.

**Fig. 1:**
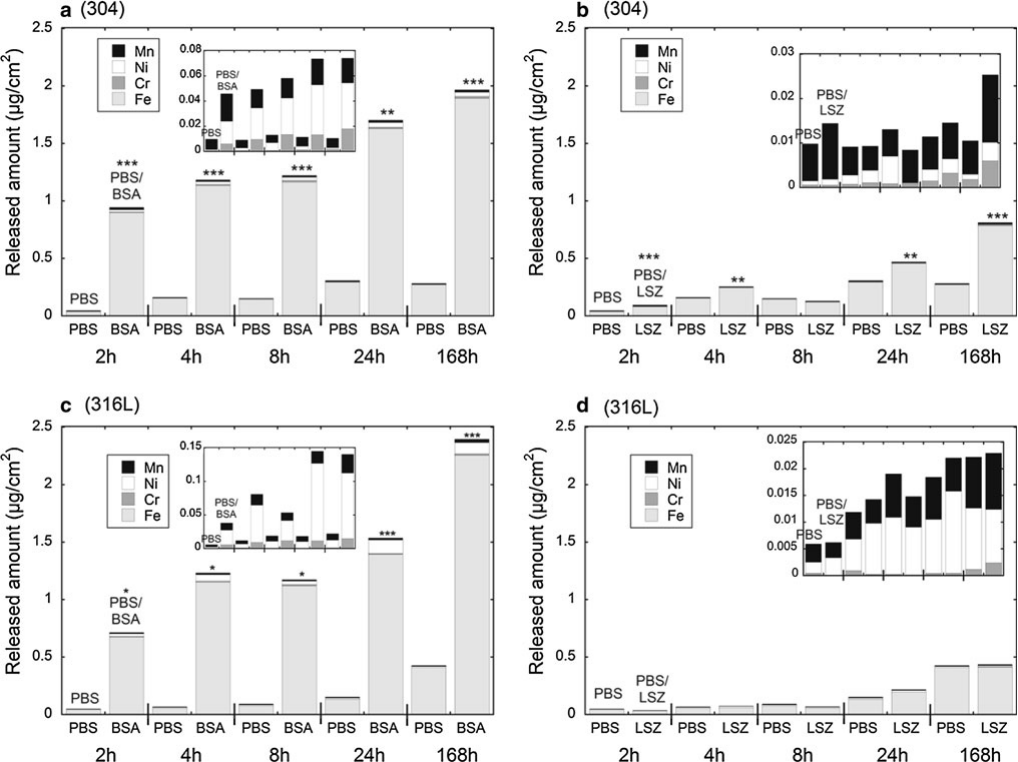
Amount (μg/cm^2^) of metals (Fe, Cr, Ni, and Mn) released from austenitic stainless steel grades 304 (**a**, **b**), and 316L (**c**, **d**) exposed up to 168 h in PBS, PBS + BSA, and PBS + LSZ. *Asterisks* indicate significant increases in total (Fe + Cr + Ni + Mn) metal release in protein solution (PBS + BSA and PBS + LSZ, respectively) compared with PBS, as calculated by a Student’s *t* test (unpaired data with unequal variance), *P < 0.05, **P < 0.01, and ***P < 0.001. The *inset* graphs are magnified with Fe excluded

The highest total released quantities were observed for the ferritic grade (430) in the presence of both proteins, whereas no clear trend between the austenitic (304, 310, 316L) and the duplex (2205) grades was evident. The ranking was different in PBS + LSZ (for 316L), compared with PBS and PBS + BSA, discussed in Sect. 3.3. Similar ranking, although less obvious, has previously been observed for the same grades exposed to Gamble’s solution, pH 7.4, and artificial lysosomal fluid, pH 4.5 [6]. Due to the similarity in released quantities/rates between the austenitic and the duplex grades, detailed kinetic studies were only conducted for the grades that generally released the largest extent of metals, i.e. grades 430, 304 and 316L, where the latter is the most relevant grade from a biomaterial perspective. Their individual time-dependence is displayed in Figs. 1 (304 and 316L) and 2 (430).

**Fig. 2:**
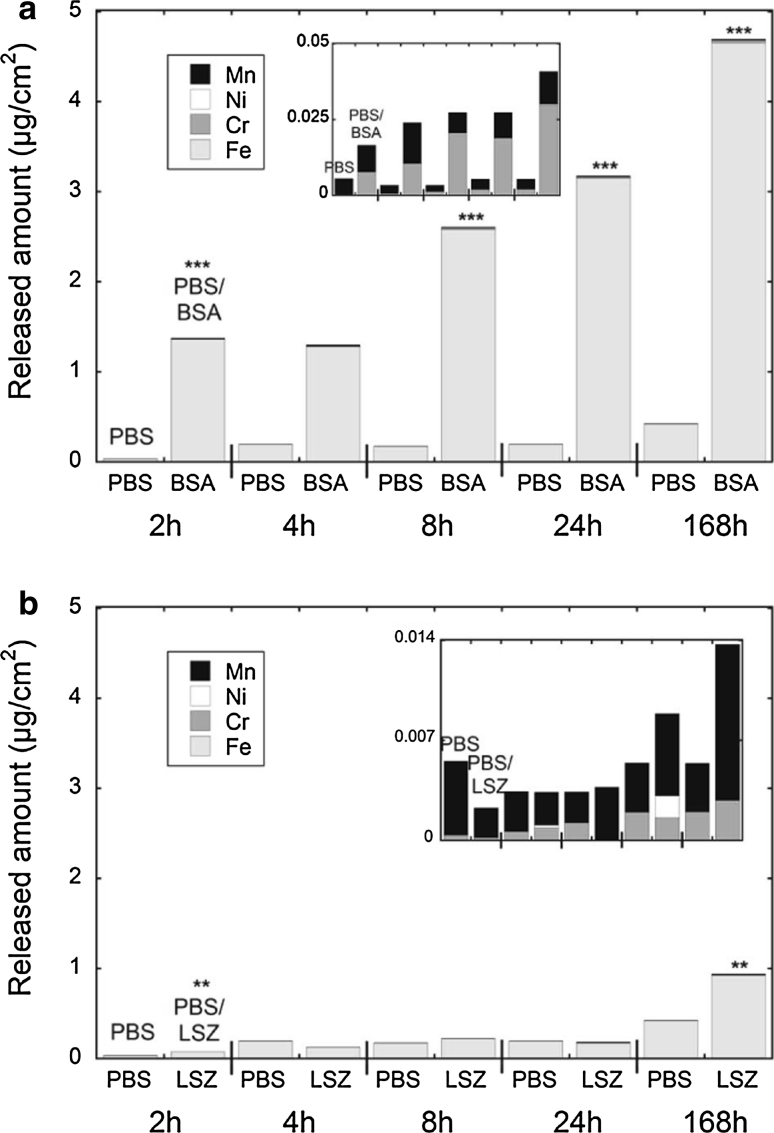
Amount (μg/cm^2^) of metals (Fe, Cr, Ni, and Mn) released from a ferritic stainless steel grade 430 (**a**, **b**) exposed up to 168 h in PBS, PBS + BSA, and PBS + LSZ. *Asterisks* indicate significant increases in total (Fe + Cr + Ni + Mn) metal release in protein solution (PBS + BSA and PBS + LSZ, respectively) compared with PBS, as calculated by a Student’s *t* test (unpaired data with unequal variance), *P < 0.05, **P < 0.01, and ***P < 0.001. The *inset* graphs are magnified with Fe excluded

**Fig. 3:**
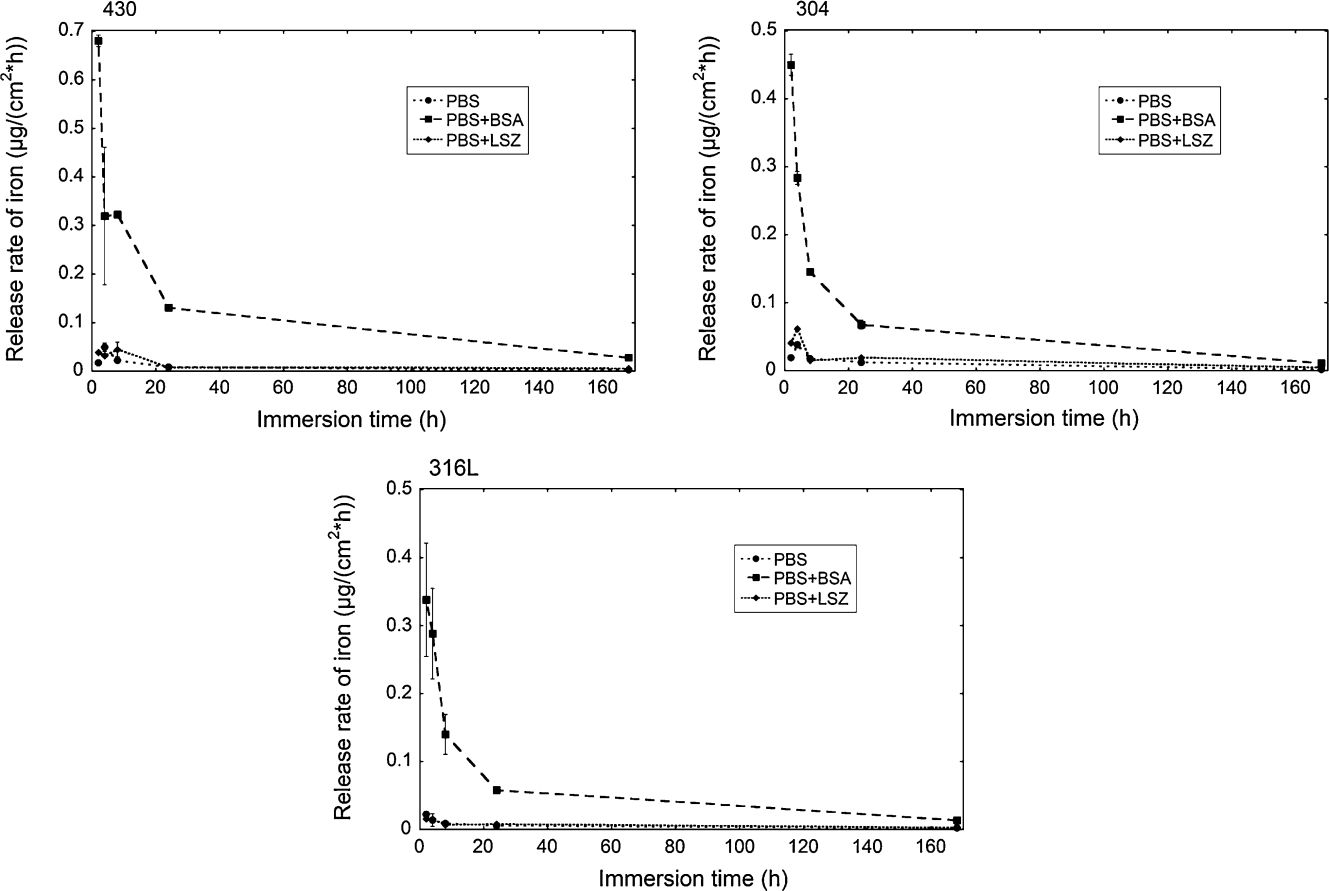
Release rates of iron from the stainless steel grades 430, 304, and 316L, expressed as μg/(cm^2^ h), for exposures in PBS, PBS + BSA, and PBS + LSZ, for up to 168 h. The *error bars* represent the standard deviation between triplicate samples (blank concentration subtracted)

The release of all metals was strongly enhanced in the presence of BSA. Iron was predominantly released for all grades and in all fluids, a well-documented, general phenomenon for stainless steels and chromium-based alloys [2, 6–9, 20, 31, 33, 38–44]. There was also a significant release of nickel, though still at relatively low concentrations in the case of austenitic stainless steels (304 and 316L, Fig. 1a, c). This is not anticipated when considering that nickel is normally not present in the outermost surface oxide of stainless steel [45] and was not detected in this study by means of XPS after exposure to protein solutions. However, a strong enhancement of released nickel has been observed in the presence of complexing agents for 316L powders [33]. The metal release rates decreased strongly with time, illustrated for iron release from grades 430, 304, and 316L in Fig. 3. Strongly decreasing metal release rates with time are typical for stainless steels in most media [6–8, 31, 38, 39, 41, 46]. This is explained by the initial dissolution and repassivation of instable phases and defects on the surface, and by the passivation of the surface, e.g. through chromium enrichment in acidic and/or complexing solutions. A small initial increase of the metal release rate with time was observed for grades 304 and 430 in PBS and PBS + LSZ, Fig. 3. This effect has not been observed in other kinetic studies of similar alloys in PBS or in complexing media [8, 41]. However, a similar time-dependence has previously been observed in highly complexing solutions such as proteins and citric acid [8]. Minor initial increased rates followed by strongly decreased metal release rates for stainless steels and other passive alloys may be related to (i) complexation-induced metal release in the case of complexing solutions [33, 47], or (ii) slowly dissolving surface defects or inclusions, followed by a repassivation of the surface oxide [8]. Observed amounts of metals released into PBS were consistent with literature findings for stainless steel 316 in Hank’s solution (pH 7.4, no protein content) [48], while the corresponding release into protein containing PBS fluid was comparable with artificial lysosomal fluid (ALF, pH 4.5) [6]. The total amount of released metals from the different stainless steel grades into PBS + BSA, i.e. grade 430 > 316L > 304, was in agreement with previous studies in ALF [6]. The amount of metals released into PBS and PBS + BSA from 316L is consistent with literature findings for longer exposure times [31].

**Fig. 4:**
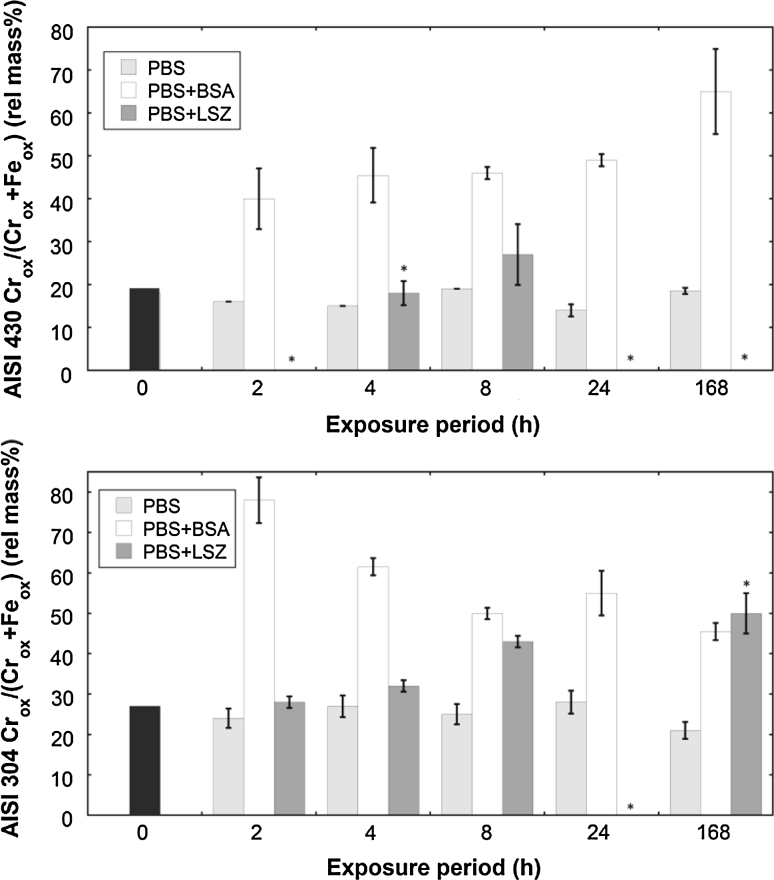
Relative mass ratio between oxidized chromium and oxidized iron (Cr_ox_/(Cr_ox_ + Fe_ox_)) in the surface oxide of unexposed and exposed grade 430 (*top*) and 304 (*bottom*) in PBS, PBS + BSA, and PBS + LSZ for exposure periods up to 168 h. The *asterisks* indicate a too dense or too thick adsorbed LSZ layer to enable XPS measurements from that replicate surface area

A significant release of iron, observed already after 2 h of exposure in PBS + BSA, Figs. 1a and 2a, correlated with a rapid and considerable enrichment of chromium in the surface oxide, Fig. 3 (430 and 304), based on XPS results. The relative surface mass ratio of oxidized chromium to oxidized chromium and iron (Cr_ox_/(Cr_ox_ + Fe_ox_)) increased from 0.27 to 0.78 (304) and from 0.18 to 0.40 (430) within the first 2 h of exposure. This mass ratio was reduced with time for 304 (to 0.46 after 168 h), but further increased (to 0.65) for 430. Similar enrichments were also evident for grade 316L exposed in PBS + BSA (c.f. Fig. 9a, discussed below), without any significant effect in PBS only, when compared to the unexposed surface. Chromium enrichment was also evident for grade 304 upon exposure in PBS + LSZ (Fig. 4), however at a significantly slower rate. Similarly, the effect of LSZ was less obvious for grade 430 (Fig. 4), although not possible to determine due to a thick surface coverage of LSZ after 24 and 168 h of exposure. A thicker layer of LSZ compared to BSA was supported by XPS data (significantly reduced substrate signals, no detection of substrate signals for several time points, Fig. 4).

Observed results for grade 304 resemble changes of the surface oxide of stainless steels upon passivation treatments with complexing acids, such as citric acid. A significantly larger chromium enrichment compared with treatments with non-complexing nitric acid has been reported for stainless steels upon citric acid passivation treatment [49, 50]. Worth noting is that the complexation capacity of surface iron atoms with BSA seems to be different for grade 304 compared with grade 430 as passivation-like surface changes were only observed for grade 304. This was reflected in differences in observed kinetics of the metal release processes for grades 304 and 430, Figs. 1 and 2. Approximately 50 % of the total amount of released metals determined after 1 week was already released after 2 h from 304 when exposed in PBS + BSA (Fig. 1a). Corresponding released amounts were only 30 % from grade 430. However, the significantly larger chromium enrichment of the surface oxide after 2 h observed for grade 304 compared with 430 was not reflected in a larger amount of released iron, Figs. 1 and 2. This suggests that iron remains on the surface, most probably complexed to some extent to adsorbed proteins.

Based on the theoretical number of nitrogen atoms per molecule of BSA (780) and LSZ (193) and a 2.5 larger surface cross-section area of BSA compared with LSZ, a given protein layer thickness would result in 1.6 times more nitrogen on the surface in the case of BSA compared with LSZ. XPS measurements of the amount of nitrogen on the surface resulted in a ratio of 0.3–0.4 between grade 304 exposed in BSA compared with LSZ, which suggest a radically thicker layer of LSZ. The strong enrichment of chromium in the mixed chromium and iron surface oxide induced by the presence of proteins, primarily BSA, resulted in a less significant enhancement of released metals with time. Similar observations have been observed for stainless steel exposed to acidic or strongly complexing solutions [51].

Table 3 compiles momentary release rates of iron into PBS and PBS + BSA for the stainless steel grades 430, 304, and 316L. In all cases (solutions and grades), the momentary release rates generally decreased with time, and to a stronger extent in PBS + BSA compared with PBS. For example the determined momentary release rates of iron in PBS + BSA between the 25th and 168th hour were only 0.4–1.8 % of the initial rate (0–2 h), while corresponding numbers were approximately 10 % in PBS. For the minor elements released, i.e. chromium, nickel, and manganese, the final momentary release rates (24–268 h) were less than 2.3 % of the initial rates (0–2 h) in all cases, both in PBS and PBS + BSA (Tables 4, 5, 6). In contrast to iron (Table 3), where the release rates were only slightly larger in PBS + BSA compared with PBS (factor 2–6, *P* < 0.009) for the time period between 24 and 168 h, the chromium release rates were still enhanced by a factor of 5 (316L, *P* = 0.05) to 1,000 (430, *P* = 0.003; non-significant for 304) in PBS + BSA compared with PBS (Table 4). However, release rates of chromium were significantly lower (>60-fold) compared with iron. Possible reasons for the significant enhancement of released chromium in PBS + BSA compared with PBS between 24 and 168 h could be the strong chromium enrichment in the surface oxide and/or slower dissolution kinetics of chromium compared with iron, by the mechanisms discussed below. In the case of nickel (Table 5), a relatively slow decrease, or even increase, of momentary released nickel were observed during the first three exposure periods. This observation was more pronounced in PBS compared with PBS + BSA. The final momentary nickel release rate (24–168 h) was very low in all cases, <1 % of the initial rate (Table 5). The difference in nickel release between PBS and PBS + BSA was strongest in the beginning (>10-fold, *P* = 0.003 for 304, non-significant for 316L), and not possible to calculate for the last time period due to very low momentary release rates (<0, for all triplicate samples), Table 5. Momentary release rates of manganese showed the strongest reduction with time compared with all metals investigated (between 50 and 98 % of the total (168 h) release within the first 2 h of exposure), Table 6. Despite the fact that manganese was not detected by means of XPS in the outermost surface oxide, it was still mostly released initially. These results are in concordance with reported observations for 316L powder particles containing significant amounts of manganese in the surface oxide [33]. The results suggest that a protein-induced enrichment of chromium in the surface oxide with time increases its stability from a metal release perspective.

**Table 3:** Momentary release rates of iron (μg/(cm^2^ h)) from stainless steel grades 430, 304 and 316L into PBS + BSA, and PBS (the release rate of the shorter time period is subtracted from that of the longer time period, e.g. for 24–168: amount average metal released at 168 h—amount average metal released at 24 h, normalized to time and surface), presented as average values between triplicate samples

Time period (h)	304 PBS	304 PBS + BSA	316L PBS	316L PBS + BSA	430 PBS	430 PBS + BSA
0–2	0.019	0.450	0.022	0.338	0.017	0.681
2–4	0.058	0.117	0.006	0.239	0.081	<0
4–8	<0	0.008	0.005	<0	<0	0.326
8–24	0.010	0.029	0.004	0.017	0.001	0.035
24–168	<0	0.002 (0.4 % of initial)	0.002 (9.1 % of initial)	0.006 (1.8 % of initial)	0.002 (11.8 % of initial)	0.011 (1.6 % of initial)

<0 indicates that the final released average amount was smaller than the initial average rate, e.g. due to small differences or precipitation processes during time

**Table 4:** Momentary release rates of chromium (μg/(cm^2^ h)) from stainless steel grades 430, 304 and 316L into PBS + BSA, and PBS (the release rate of the shorter time period is subtracted from that the rate of the longer time period), presented as average values between three replicate samples

Time period (h)	304 PBS	304 PBS + BSA	316L PBS	316L PBS + BSA	430 PBS	430 PBS + BSA
0–2	3.02E−4	0.0030	2.24E−4	0.0028	1.92E−4	0.0039
2–4	7.68E−5	0.0018	2.41E−4	0.0018	1.19E−4	0.0014
4–8	3.11E−5	0.0010	<0	0.0006	1.56E−4	0.0025
8–24	3.91E−5	<0	1.06E−5	<0	4.64E−5	<0
24–168	2.42E−6 (0.8 % of initial)	3.31E−5 (1.1 % of initial)	5.07E−6 (2.3 % of initial)	2.26E−5 (0.8 % of initial)	7.24E−8 (0.003 % of initial)	7.58E−5 (2.0 % of initial)

<0 indicates that the final released average amount was smaller than the initial average rate, e.g. due to small differences or precipitation processes during time

**Table 5:** Momentary release rates of nickel (μg/(cm^2^ h)) from stainless steel grades 430, 304 and 316L into PBS + BSA, and PBS (the release rate of the shorter time period is subtracted from that of the longer time period), presented as average values between three replicate samples

Time period (h)	304 PBS	304 PBS + BSA	316L PBS	316L PBS + BSA	430 PBS	430 PBS + BSA
0–2	0.0005	0.0090	0.0010	0.0109	0	0
2–4	0.0006	0.0035	0.0019	0.0168	0	0
4–8	0.0010	0.0010	0.0012	<0	0	0
8–24	<0	0.0007	<0	0.0053	0	0
24–168	<0	<0	9.77E−6 (1 % of initial)	<0	0	2.12E−6

<0 indicates that the final released average amount was smaller than the initial average rate, e.g. due to small differences or precipitation processes during time

**Table 6:** Momentary release rates of manganese (μg/(cm^2^ h)) from stainless steel grades 430, 304 and 316L into PBS + BSA, and PBS (the release rate of the shorter time period is subtracted from that of the longer time period), presented as average values between three replicate samples

Time period (h)	304 PBS	304 PBS + BSA	316L PBS	316L PBS + BSA	430 PBS	430 PBS + BSA
0–2	0.0042	0.0109	0.0017	0.0053	0.0026	0.0044
2–4	<0	<0	0.0008	0.0027	<0	<0
4–8	<0	0.0002	0.0008	<0	<0	<0
8–24	8.25E−5	0.0003	<0	0.0004	8.14E−5	0.0001
24–168	1.1E−6 (0.03 % of initial)	<0	1.15E−5 (0.7 % of initial)	6.91E−5 (1.3 % of initital)	<0	1.58E−5 (0.4 % of initial)

“<0” indicates that the final released average amount was smaller than the initial average rate, e.g. due to small differences or precipitation processes during time

As previously found in several studies for stainless steels [7, 31, 42–44, 46, 51], the metal release was neither proportional to the bulk nor to the surface oxide composition, illustrated in Tables S1, S2 (supplementary material, amount of metal released normalized to corresponding content in the bulk alloy and surface oxide, respectively). Normalized to the nominal bulk composition (Table 1), the release of metals followed the general order: Mn ≈ Fe ≫ Ni > Cr for austenitic stainless steels (304 and 316L), results in agreement with [33]. The corresponding sequence for ferritic stainless steel (430) was Mn ≈ Fe ≫ Cr ≫ Ni, Table S1. Normalized to the surface oxide content, the release of metals followed a different general order with Mn (>Ni, only for austenitic stainless steels) ≫ Fe ≫ Cr, since neither nickel nor manganese were observed in the uppermost surface oxide, and iron was released preferentially.

#### Electrochemical aspects

**Fig. 5:**
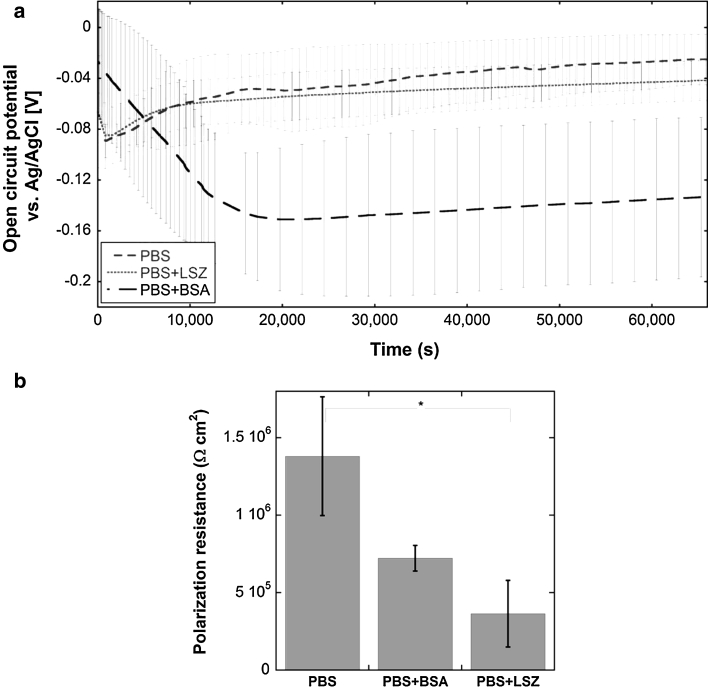
**a** Open circuit potential over time for stainless steel 304 exposed in PBS, PBS + LSZ, and PBS + BSA. The *error bars* shown indicate the standard deviation between two replicate measurements. **b** Polarization (corrosion) resistance (Ω cm^2^) of stainless steel grade 304 in PBS, PBS + BSA, and PBS + LSZ after 18 h of exposure (*error bars*: standard deviation between three replicate measurements)

Time-dependent surface processes were also evident from open-circuit measurements in PBS and PBS + BSA over time (up to 18 h), illustrated in Fig. 5a for grade 304. The OCP in PBS was relatively constant during the measurement up to 18 h (with a slightly increasing trend), an effect not observed in the protein-containing solution. In PBS + BSA, OCP was initially slightly higher (non-significant, *P* > 0.05) compared to conditions in PBS only, however the passive properties were gradually reduced over time and reached approximately similar levels after 60 min, Fig. 5a. OCP decreased further with time to significant lower values compared with observations in PBS and reached a relatively constant potential (−0.16 V) after approximately 8 h that slightly increased to −0.13 V up to 18 h (Fig. 5a). OCP measurements of grade 304 in PBS + LSZ were, in contrast to findings in PBS + BSA, very similar compared with PBS, Fig. 5a. A similar OCP—time dependence has previously been reported for a magnesium rare-earth alloy in protein solutions (40 g/L albumin in a simulated body fluid) [52]. A similar OCP-time dependence was also reported for stainless steel grades 304 and 316L exposed in Hank’s solution with and without proteins [53], and for grade 316L in PBS of different BSA concentration [54].

In concordance with the literature [28, 53], the exposure of grade 304 in PBS + BSA resulted in a reduced polarization resistance, i.e. higher corrosion rates, compared with the non-protein containing solution, Fig. 5b, measured after 18 h exposure. Similar observations were made for grade 304 exposed in PBS + LSZ compared to PBS only, Fig. 5b. This reduction in polarization resistance was slightly larger compared with observations in PBS + BSA, an effect possibly explained by the rapid initial passivation of 304 upon exposure in BSA (Fig. 3) as discussed in Sects. 3.1.1 and 3.3. These findings show that the metal release process, based on the extent of released metal measured in solution, is not solely a result of an electrochemical process at these conditions and that the extent of metal release cannot be predicted from polarization (corrosion) resistance data only.

#### Effect of protein adsorption

**Fig. 6:**
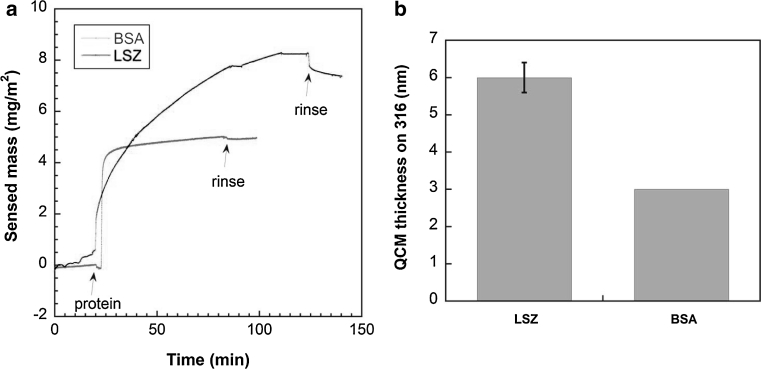
Sensed mass (mg/m^2^) of adsorbed LSZ and BSA on stainless steel grade 316 determined by means of QCM (**a**). The *arrows* indicate the time point of protein introduction and rinsing (with PBS). Corresponding QCM layer thicknesses according to Voigt calculations for LSZ and BSA adsorption on 316 substrates in PBS solution of pH 7.4 (**b**). *Error bars* show the deviation between two independent measurements (only one measurement for BSA). *Note* adsorption measurements were performed using lower protein concentrations (BSA—10 times, LSZ—2 times) compared with the metal release investigation, as in [20]. Details on Voigt modeling and reproducibility are given in [20] for chromium substrates compared with 316 and other substrates (Color figure online)

Previous in-depth adsorption studies on chromium metal sputtered QCM crystals by the authors have shown BSA to adsorb with monolayer coverage already within a few minutes [20]. In that study, the adsorption of LSZ continued to increase with time, i.e. several layers were adsorbed on the surface, and no adsorption plateau was observed after 1 h, when exposed to PBS at pH 7.4 (and at pH 4) [20]. Similar observations and very slow desorption of both BSA and LSZ, indicative of strong surface-protein bonding were reported by the authors for grade 316 sputtered QCM crystals [20]. The calculated layer thicknesses, according the calculations explained in [20], and recorded changes in protein mass over time for adsorbed LSZ and BSA (in PBS at pH 7.4) are compiled in Fig. 6 and reveal a thicker layer for LSZ (6 nm after 1 h) compared with BSA (3 nm after 1 h) on grade 316. The comparison with the molecular dimensions of LSZ in solution (4.5 × 3 × 3 nm^3^ [22]) and BSA (8 × 8 × 3 nm^3^ [55]) suggests LSZ to adsorb with more than a monolayer coverage, independent of surface orientation (side-on or end-on), and BSA to adsorb as a side-on monolayer. These findings are in line with XPS findings and with adsorption studies of BSA and LSZ onto chromium metal [20] and other surfaces previously reported [16, 17, 19].

**Fig. 7:**
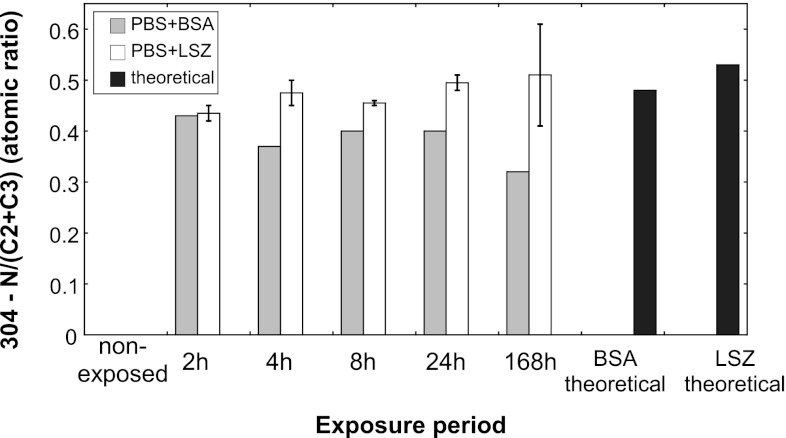
Relative atomic ratio between nitrogen and oxidized carbon on unexposed and exposed 304 stainless steel in PBS, PBS + BSA and PBS + LSZ

Adsorption of both proteins was confirmed by ex-situ XPS investigations, illustrated for grade 304 in Fig. 7. The nitrogen (N 1s) peak at 399.8 ± 0.3 eV was attributed to amine and amide species [56]. The carbon peak (C 1s) was resolved into three peaks at 285 eV (C1: C–C, C–H bonds), corresponding to adventitious carbon, at 286.5 ± 0.2 eV (C2: C–N and C–O bonds) and at 288.1 ± 0.1 eV (C3: C=C–O (carboxyl) and O=C–N (amide) groups) [34]. Representative C 1s and N 1s core spectra are displayed in Fig. S1. A nitrogen to carbon atomic ratio excluding adventitious carbon (N/(C2 + C3)) of 0.43 was determined for grade 304 in PBS + BSA already within 2 h of exposure. This ratio correlated relatively well with the observed ratio for BSA powder (0.48) [56] and with the theoretical calculation from the amino acid sequence for BSA (0.48) [57]. The slightly lower value reflects an additional contribution of oxidized carbon contaminants (in C2 and C3) present on the surface. The measured ratio was 0.38 ± 0.04 for the different time periods. Higher nitrogen to oxidized carbon atomic ratios of 0.47 ± 0.06 were observed for grade 304 exposed in PBS + LSZ, similar to the theoretical value of 0.53 calculated from the amino acid sequence of hen egg LSZ [58]. Similar atomic ratios of nitrogen to oxidized carbon were determined for grades 430 (BSA: 0.37 ± 0.05; LSZ: 0.45 ± 0.03) and 316L (BSA: 0.41 ± 0.02).

### Protein-induced metal release from stainless steels: effect of protein concentration

**Fig. 8:**
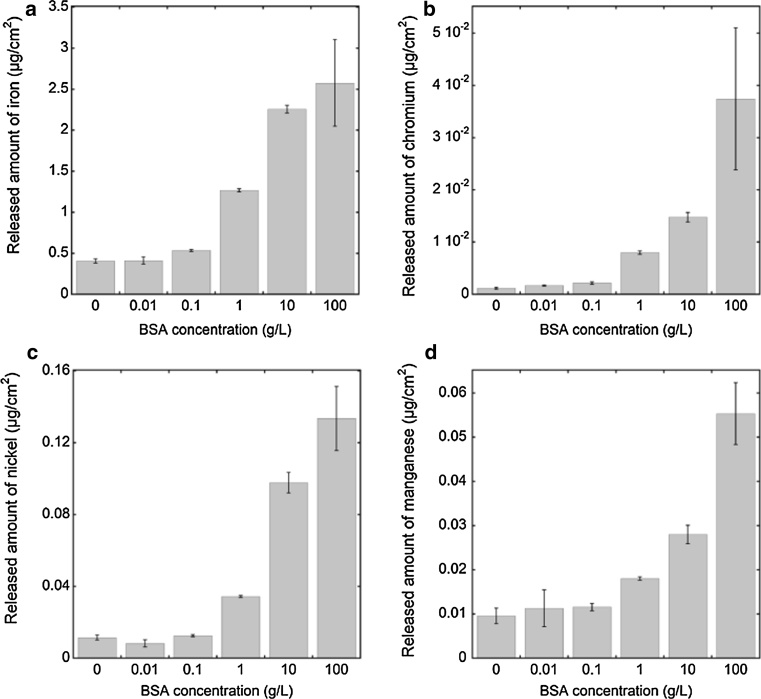
Amount of metal release in μg/cm^2^ for different BSA concentrations in PBS for 316L after 168 h of exposure: **a** iron release, **b** chromium release, **c** nickel release, **d** manganese release

**Fig. 9:**
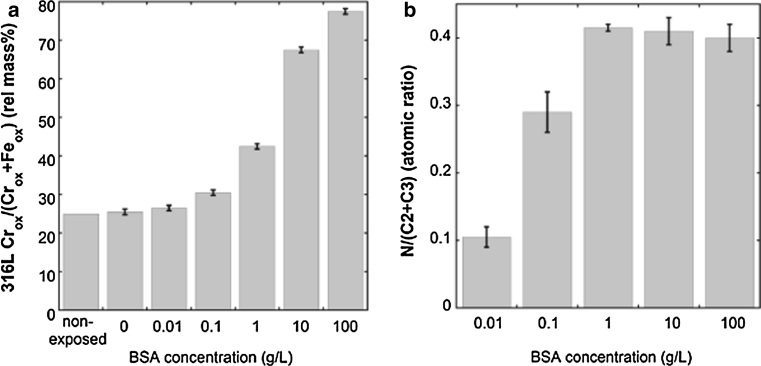
**a** Amount of oxidized chromium per amount of oxidized chromium and iron (relative mass%) and **b** relative atomic ratio between nitrogen and oxidized carbon, measured by means of XPS; all for 316L unexposed and after exposure to solutions of PBS with different BSA concentrations, for 168 h respectively

The effect of protein concentration in solution on the released amount of metals was investigated using different BSA concentrations (ranging from 0.01 to 100 g/L BSA) in PBS during 168 h for grade 316L combined with ex-situ surface compositional measurements, Figs. 8 and 9. Non-significant (*P* > 0.05) changes compared to non-protein containing PBS solutions were observed both from a surface oxide (Cr_ox_/(Cr_ox_ + Fe_ox_)) and metal release perspective up to a concentration of 0.1 g/L BSA in PBS, with the exception of chromium release (significant, *P* < 0.05, increase already at 0.01 g/L BSA) and iron release (significant already at 0.1 g/L BSA). The adsorption of BSA on the surface appeared to depend on the protein concentration in solution, as indicated by the nitrogen to oxidized carbon ratio measured by XPS, Fig. 9b (assuming that surface contamination contributes to a larger extent to the C_ox_ signal at lower protein concentrations). This may be related to a gradually more dense and rigid close-packed monolayer coverage of BSA. At a BSA concentration of 1 g/L, the total amount of released metals was enhanced (approximately by a factor of 2, Fig. 8, *P* < 0.05) as well as the corresponding relative chromium content of the surface oxide (from an average Cr_ox_/(Cr_ox_ + Fe_ox_) mass ratio of 0.33 ± 0.03 in 0.1 g BSA/L to 0.44 ± 0.01, Fig. 9a, *P* < 0.05). This suggests complexation of iron atoms on the surface with adsorbed proteins. Another 10-fold increase of the protein concentration to 10 g/L showed similar trends with significantly (*P* < 0.05) enhanced amounts of released metals, in particular pronounced for nickel, Fig. 8c, and a substantial enrichment of chromium in the surface oxide (to a Cr_ox_/(Cr_ox_ + Fe_ox_) mass ratio of 0.78 ± 0.04, *P* < 0.05), Fig. 9a. These findings could be related to both further complexation of iron surface atoms and/or to local defects in the surface oxide induced by the complexation and/or local lowering of surface pH, discussed in detail in Sect. 3.3. It could in particular explain the release of nickel, present in a metal surface layer adjacent the uppermost surface oxide [4, 45, 59]. A ten times higher protein concentration, 100 g/L, induced a less significant increase of released metals (*P* > 0.06) and enrichment of chromium in the surface oxide (*P* = 0.005), however, still significant for manganese (*P* = 0.04), Fig. 9d. Reasons for the less significant increase, especially in the case of iron release, could be attributed to its relatively high concentration in solution (compared to chromium and manganese) and an achieved equilibrium suppressing further dissolution. The 100 g/L BSA + PBS solution was experimentally challenging to handle prior to analysis since viscous solutions (gel-like) were formed with time in the acidified samples that were difficult to dissolve and analyze (the reason for the relatively large error bars). These results should hence not be over-interpreted. Previous QCM studies by the authors on HSA adsorption on chromium metal revealed no further increase of the sensed mass at protein solution concentration exceeding 0.5 g/L HSA [20], a mass corresponding to one full monolayer coverage. According to the adsorption studies in this study for 316, BSA adsorbed with a monolayer coverage or less within 1 h of exposure in PBS + BSA (1 g/L), Fig. 6. These findings suggest that a full monolayer coverage of BSA is required to both significantly enhance the release of metals and result in a concomitant enrichment of chromium in the surface oxide compared with protein-free solution. A further increase of the protein concentration in solution results in increased metal release, suggesting that, beside the adsorbed protein layer, also solution proteins and/or the exchange of adsorbed proteins are important aspects governing the metal release process.

### Protein-induced metal release from stainless steels: possible mechanisms

#### Change of pH and ion concentration at the surface

**Fig. 10:**
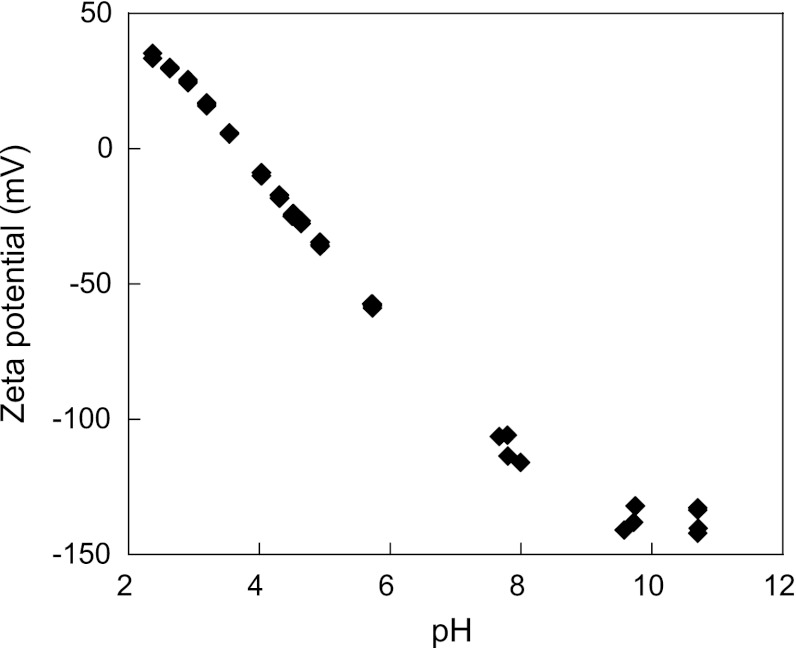
Zeta potential (ZP) values of a 316 QCM surface measured in a 1 mM KCl solution as a function of solution pH

In order to study the local surface environment (charge and pH), the surface charge of a 316 QCM crystal was determined (Fig. 10). These measurements revealed a zeta potential very close to zero in the pH interval between 3 and 4 in a 1 mM KCl solution and an approximate zeta potential of −100 mV at pH 7.4. These observations are in concordance with other studies that report a negative zeta potential at pH 6.0 for an evaporated stainless steel surface [60] and an apparent surface potential of −85 mV at pH 5.8 for an evaporated chromium metal crystal in a 1 mM NaCl solution [20]. Protonation of the hydroxylated surface oxide will result in the weakening of bonds of surface atoms and subsequent detachment of metal ions or ionic complexes [61]. Charge regulation of the protein surface (combined with protein anisotropy) has recently been theoretically shown to explain protein adsorption onto similar charged surfaces [62], which is the case for the BSA adsorption on stainless steel in this study. The proton concentration at the surface using the Boltzmann distribution for species A is given by Eq. 1:

1 $$  \left[ {{\text{A}}^{ + } } \right]_{{\text{s}}}  = \left[ {{\text{A}}^{ + } } \right]_{{\text{s}}} \exp \left( {\frac{{ze{{\Uppsi }}_{0} }}{{k_{B} T}}} \right) $$

where the subscript “*s*” indicates the surface, and *z*, *e*, *k*_B_ *, T*, and Ψ_0_ denote the valence of the ion, the elementary charge, the Boltzmann constant, the absolute temperature, and the electrostatic potential at the surface of the potential determining ion, respectively. When considering the proton concentration at the surface, it is evident from Eq. (1) that the surface pH differs from the bulk pH (unless Ψ_0_ = 0). This is also the case for protein-solution interfaces where the bulk pH is, in general, quite different compared to a pH near a protein surface [63]. For a zeta potential of −100 mV at a bulk pH of 7.4, a significant reduction of the pH (5.8) would be obtained, however, this number is most probably underestimated due to the higher ionic strength in the PBS buffer (0.15 M NaCl). Assuming the surface potential to be lowered to about −20 mV due to adsorption of counter-ions at the surface, the pH at the surface would only be reduced to 7.1.

Different an- and cations, Na^+^, K^+^, Cl^−^, HPO_4_^2−^, H_2_PO_4_^−^, and OH^−^, exist in the PBS buffer. At a surface potential of −20 mV, Na^+^ is still enriched at the surface, compared with the bulk solution (to 0.35 M, compared with 0.164 M in PBS), and chlorides are depleted (0.07 M on surface compared with 0.15 M in PBS). However, the ions in the PBS buffer are also co- and counter-ions for the proteins. The adsorption of net positively charged LSZ will lower the negative surface potential of the stainless steel surface and thereby increase the concentration of chloride at the interface. For example, decreasing the surface potential from −100 to −20 mV increases the chloride concentration at the stainless steel surface from 3.5 to 70 mM according to Eq. (1). In contrast, the counter-ions of the negatively charged BSA, H^+^ and Na^+^, would be enriched at the interface between the surface and protein. This might reduce the surface pH even further. In the case of LSZ, the counter-ion is mainly Cl^−^. Adsorption of LSZ would hence result in a higher chloride concentration at the stainless steel surface. When considering the corrosion resistance to chlorides (pitting corrosion resistance) of the different stainless steel grades, 316L is known to be most pitting corrosion resistant grade among the grades investigated due to its molybdenum content [64–66]. This might be one explanation for its different ranking among the stainless steel grades when exposed in the LSZ solution, as LSZ may induce pitting corrosion events by the attraction of chlorides for the less pitting corrosion resistant grades only (all except 316L).

Literature findings on the pH-dependence on the metal release process for different stainless steel grades (Table S3, supplementary material) and pH- and solution-dependent enrichments of chromium in the outermost surface oxide (Table S4, supplementary material) suggest that a lowering of the surface pH induced by BSA is not the only factor that can explain the enhancement of released metals, possibly with exception of manganese release, and that a reduced surface pH to approx. pH 4.3 or less would be necessary to induce observed effects. However, especially in the case of nickel and manganese release, not present in the outermost surface oxide, and in the case of nickel release that was slightly postponed (Table 4), a locally reduced surface pH (BSA) and/or local adsorption and enrichment of chlorides (LSZ), may be of higher importance in comparison to the release of chromium and iron.

#### Surface complexation between protein and metal atoms

Another important explanation for the reduction of the polarization resistance (in the presence of both BSA or LSZ), the enrichment in chromium in the surface oxide (BSA and LSZ), and the enhancement of released metals (mostly by BSA) is the preferential formation of iron-protein surface complexes, in favor of chromium-complexes, and hence an increased release of iron both when exposed to BSA or LSZ. The formation of metal-protein surface complexes and the detachment of any metal complexes or metal ions may depend on several factors such as (i) adsorption mechanisms of the proteins, (ii) metal atom availability in the surface oxide and its changes with time, (iii) oxide stability, and (iv) stability constants of protein surface groups and metal ions. Previous QCM studies by the authors have shown both BSA and LSZ in PBS to readily adsorb on chromium metal and stainless steel 316 surfaces [20] with sensed masses corresponding to a monolayer coverage for BSA and thicker layers for LSZ (Fig. 6). Metal atom availability for any protein-metal surface complex strongly depends on the surface characteristics and the microstructure of the stainless steel. It is well known that a mixed iron and chromium oxide is present in the outermost surface, as also confirmed by this study, while nickel is enriched in a layer adjacent this surface oxide and hence not directly available at the uppermost surface without the presence of surface defects [4, 14, 45, 59]. The stabilities of chromium and iron oxides are very different and also pH dependent. At acidic, neutral or weakly alkaline pH, Fe_2_O_3_ is more soluble compared with Cr_2_O_3_ [38, 39]. However synergistic and/or antagonistic effects in the mixed oxide layer may result in a completely different dissolution behavior. Stability constants in solution of different amino acids and complexing agents (ethylenediamine-tetraacetic acid, iminodiacetic acid, glycine, and cysteine) with ions of nickel, iron, chromium and manganese suggest that complexation to trivalent iron in solution is more favorable compared with trivalent chromium, and that nickel forms the most stable complexes, followed by iron. Manganese forms the least stable complexes among the bivalent metals [67]. Complexation studies by the authors have shown chromium(III) ions to form weak complexes with proteins in solution, processes strongly dependent on size and protein concentration [68]. In the case of complexing agents, ligand-promoted dissolution of metal oxides has been described in the literature and the detachment step has been identified as the rate-limiting step [69]. It is worth noting that the complexation ability of the surface oxides seems to be different for different stainless steel grades or surfaces. Results of this study imply complexation of iron atoms with BSA at the surface oxide of stainless steel grade 304 already within 2 h of exposure to PBS + BSA, an exposure that resulted in a significant enrichment of chromium in the surface oxide. This resulted in a passivation-like behavior with strongly decreased metal release rates (compared to other grades) and a significantly increased polarization resistance (compared with PBS + LSZ) for grade 304. Previous findings have shown very different complexation abilities of 316L stainless steel powder particles of different size, effects partially attributed to differences in surface oxide composition and microstructure [33]. A recent surface analytical study at similar conditions as in this study suggests that BSA and LSZ are chemisorbed rather than physisorbed on the 316L surface [14].

#### Importance of mechanisms for metal release from stainless steels and corrosion

Preferential complexation to iron in favor of chromium and the formation of complexes between proteins and iron might explain the decreased polarization resistance (Fig. 5) and the strong chromium enrichment in the surface oxide (Figs. 4, 9a (BSA only)). The time dependence in OCP observed in PBS + BSA (Fig. 5) furthermore suggests the involvement of more than one process and that the predominant process responsible for a significantly reduced OCP determined in PBS + BSA compared with PBS only, after approximately 3 h, is initially slow, as complexation might be. In contrast, protein adsorption is relatively rapid and might explain the initially slightly higher OCP in protein solutions compared with non-protein containing PBS.

In concordance with the discussion on the importance of decreased surface pH induced by BSA (Sect. 3.3.1) where a lowering of the surface pH was proposed to be mostly important for manganese, and less important for iron, nickel, and chromium, complexation is most favorable for iron, nickel, and chromium, and least pronounced in the case of manganese. Previous investigations on the importance of the solution complexation capacity on released iron, chromium, nickel, and manganese from 316L powders [33] showed that the manganese release was least dependent on complexation mechanisms and most on the solution pH. The substantially higher metal release in PBS + BSA compared with PBS + LSZ might be explained by (i) the lack of capacity of LSZ, being positively charged at pH 7.4, to lower the local surface pH, (ii) its adsorption as a thicker layer (as indicated by QCM and XPS findings, Figs. 6 and 4, respectively) and its positive charge which may hinder ionic metal complexes or (positively charged) metal ions to enter the solution, (iii) slower kinetics of adsorption of LSZ compared with BSA, and/or (iv) its lower complexation capacity, as indicated by Fig. 4. Previously, a lower chromium complexation capacity was observed for LSZ compared with BSA at a given molar concentration [68]. However, at a given mass concentration, LSZ revealed a higher complexation capacity to chromium compared with BSA [68]. In this study, a mass concentration of 2.2 g/L LSZ induced no significant enhancement of released metals compared with PBS for 316L (Fig. 1d), while already a mass concentration of 1 g/L BSA induced a significant enhancement of released metals from 316L (Fig. 8). This suggests that the complexation capacity of the proteins is not the sole factor for any induced enhancement of released metals. No indications of pitting corrosion, i.e. no metastable or stable breakdowns in the OCP measurements, as discussed in [70] for 316L powders, were evident in this study. Any pitting corrosion would furthermore result in significantly higher metal release and orders of magnitude lower polarization resistance. It can however not be excluded that small pitting events occur for the less pitting corrosion resistant grades after longer time periods, i.e. longer than 18 h (the time for the OCP measurements) due to chloride enrichment induced by LSZ (c.f. Sect. 3.3.1). It might also be possible that a chloride enrichment at the surface in the case of LSZ contributes to the lower corrosion resistance and/or reduced release of metals to a stronger extent at other test conditions than given in this study.

### Protein-induced metal release from stainless steel compared with iron metal

For comparative reasons, the effects of proteins on the extent of released iron and on the polarization resistance for iron metal sheet were investigated. Significantly higher released quantities of iron from iron metal sheet compared to iron released from stainless steel was attributed to the lack of passive properties of the surface oxide formed on iron. Opposite results were obtained compared with stainless steel (Figs. 1, 2, 5) with significantly suppressed released amounts of iron and a non-significant enhancement of the polarization resistance for the highly corroding iron metal in the presence of BSA compared to PBS only, observations in agreement with literature findings [27]:

Release of iron after 24 h (μg/cm²): 122 ± 14 (PBS) and 40 ± 1.5 (PBS + BSA);
Polarization resistance (Ω cm²): 18,800 ± 2,800 (PBS) and 29,900 ± 13,700 (PBS + BSA).

This reduced amount of released iron correlated with an improved polarization resistance, still though substantially lower compared with stainless steel, Figs. 1, 2, 5. Iron metal corrodes significantly faster (a factor of 1000 in iron release after 24 h of exposure in PBS, *P* < 0.05) compared with stainless steel and the oxide formed has substantially poorer barrier properties. Corrosion of iron is controlled by the availability of oxygen at cathodic sites at these conditions (aerated solution, pH 7.4) [71]. Proteins are proposed to partially block these cathodic sites (where oxygen reduction occurs), hence reducing the iron release rates and improving the corrosion resistance. This is in contrast with stainless steel grades that are negatively influenced by the proteins due to their very low corrosion rates.

## Summary

Both BSA and LSZ (in PBS solution, pH 7.4) enhanced the extent of released metals (Fe, Cr, Ni, and Mn), measured in solution, from all investigated stainless steel grades, with exception of 316L for LSZ. This enhancement was more pronounced for BSA compared with LSZ. Exposure in both BSA and LSZ induced a strong enrichment of chromium in the surface oxides of the stainless steel grades investigated (304, 430, and 316L). The extent was less and the kinetics slower in LSZ compared with BSA. Both BSA and LSZ reduced the polarization resistance for grade 304. This effect was more pronounced upon exposure in LSZ compared with BSA (after 18 h), due to an initial passivation of the surface oxide induced by BSA. BSA was adsorbed in a monolayer coverage, as opposed to LSZ that was adsorbed in a thicker layer. Protein solution concentration dependent measurements suggested that a full monolayer was needed to significantly enhance the release of metals and induce an enrichment of chromium in the surface oxide. After reaching a full monolayer coverage, an increase in solution protein concentration still significantly increased the metal release, possibly due to solution complexation of metal ions and/or exchange of adsorbed proteins complexed to metal ions. BSA and LSZ strongly interacted with the stainless steel surface as judged from a significant enrichment of chromium in the surface oxide. This enrichment was induced by both proteins and the OCP—time dependence for 304 in PBS + BSA. Several mechanisms were proposed to explain the capacity of proteins to enhance the release of metals from stainless steels. A lowering of the surface pH was proposed to possibly be important for the release of manganese, and less important for iron, nickel, and chromium for which complexation was more favorable compared with manganese. The enrichment of chromium in the surface oxide and the complexation capacities of the proteins suggested also complexation mechanisms to be important. Due to the chromium enrichment of the passive surface oxide in the presence of proteins, the enhancement of released metals was gradually reduced with time. Iron metal showed the reverse situation, compared with stainless steels, with slightly reduced corrosion rates and a suppressed extent of released iron due to protein blocking of cathodic sites as a result of protein-surface interactions, although still at significantly higher levels compared with stainless steels.

Contradictory information exists in the scientific literature on if and how proteins induce an enhanced release of metals and/or enhanced corrosion. This study has tried to answer some of these open questions. Further studies are though still required to understand these complex systems, since both the surface characteristics and adsorbed proteins dynamically change over time. Many different mechanisms seem to take place, and may at certain circumstances result in e.g. failure of an implant, toxic responses by the body, and/or the termination of an initiated corrosion reaction.

## Electronic supplementary material

Below is the link to the electronic supplementary material.

Supplementary material 1 (DOCX 240 kb)
