# Supplementary material for: Surface-protein interactions on different stainless steel grades: effects of protein adsorption, surface changes and metal release
Source: J Mater Sci Mater Med. 2013 Feb 2;24(4):1015–33. doi: 10.1007/s10856-013-4859-8 (PMC3620448; doi:10.1007/s10856-013-4859-8)
Supplement: Supplementary file 1 — Supplementary material 1 (DOCX 240 kb) [file 10856_2013_4859_MOESM1_ESM.docx]

**Supplementary information**

***Surface-protein interactions on different stainless steel grades – effects of protein adsorption, surface changes and metal release***

Y. Hedberg, X. Wang, J. Hedberg, M. Lundin, E. Blomberg, and I. Odnevall Wallinder

Tables S1-S4 and Figure S1

*Table S1. Release of iron, chromium, nickel, and manganese normalized to their corresponding bulk alloy content (Table 1) [µg/cm^2^/bulk] from stainless steel grades 430, 304, and 316L into PBS, PBS+BSA, and PBS+LSZ, presented as average values between 3 replicate samples*

| **Grade** | **Element** | **Exposure period / h** | **PBS** | **PBS+BSA** | **PBS+LSZ** |
| --- | --- | --- | --- | --- | --- |
| **430** | **Fe** | **2** | 0.042 | 1.64 | 0.094 |
|  |  | **168** | 0.51 | 5.62 | 1.12 |
|  | **Cr** | **2** | 0.0024 | 0.048 | 0.0012 |
|  |  | **168** | 0.012 | 0.19 | 0.018 |
|  | **Ni** | **2** | <LOD | <LOD | <LOD |
|  |  | **168** | <LOD | 0.14 | <LOD |
|  | **Mn** | **2** | 1.03 | 1.77 | 0.42 |
|  |  | **168** | 0.69 | 2.16 | 2.18 |
| **304** | **Fe** | **2** | 0.054 | 1.27 | 0.11 |
|  |  | **168** | 0.38 | 2.66 | 1.11 |
|  | **Cr** | **2** | 0.0033 | 0.033 | 0.0031 |
|  |  | **168** | 0.010 | 0.099 | 0.033 |
|  | **Ni** | **2** | 0.010 | 0.20 | 0.015 |
|  |  | **168** | 0.013 | 0.41 | 0.047 |
|  | **Mn** | **2** | 0.76 | 1.99 | 1.15 |
|  |  | **168** | 0.68 | 1.795 | 1.38 |
| **316L** | **Fe** | **2** | 0.064 | 0.98 | 0.046 |
|  |  | **168** | 0.59 | 3.26 | 0.60 |
|  | **Cr** | **2** | 0.0027 | 0.034 | 0.00050 |
|  |  | **168** | 0.0070 | 0.089 | 0.014 |
|  | **Ni** | **2** | 0.019 | 0.21 | 0.031 |
|  |  | **168** | 0.11 | 0.92 | 0.095 |
|  | **Mn** | **2** | 0.34 | 1.064 | 0.29 |
|  |  | **168** | 0.96 | 2.80 | 1.05 |

*<LOD – below limit of detection*

*Table S2. Release of iron, chromium, nickel, and manganese normalized to their corresponding surface oxide content (Figs. 4 and 8a) [µg/cm^2^/surface] from stainless steel grades 430, 304, and 316L into PBS, PBS+BSA, and PBS+LSZ, presented as average values between 3 replicate samples*

| **Grade** | **Element** | **Exposure period / h** | **PBS** | **PBS+BSA** | **PBS+LSZ** |
| --- | --- | --- | --- | --- | --- |
| **430** | **Fe** | **2** | 0.042 | 2.00 | ∞ |
|  |  | **168** | 0.51 | 11.4 | ∞ |
|  | **Cr** | **2** | 0.0022 | 0.024 | ∞ |
|  |  | **168** | 0.011 | 0.051 | ∞ |
|  | **Ni** | **2** | <LOD | <LOD | <LOD |
|  |  | **168** | <LOD | ∞ | <LOD |
|  | **Mn** | **2** | ∞ | ∞ | ∞ |
|  |  | **168** | ∞ | ∞ | ∞ |
| **304** | **Fe** | **2** | 0.050 | 2.7 | 0.11 |
|  |  | **168** | 0.34 | 3.16 | 1.51 |
|  | **Cr** | **2** | 0.0026 | 0.0089 | 0.0021 |
|  |  | **168** | 0.0098 | 0.045 | 0.012 |
|  | **Ni** | **2** | ∞ | ∞ | ∞ |
|  |  | **168** | ∞ | ∞ | ∞ |
|  | **Mn** | **2** | ∞ | ∞ | ∞ |
|  |  | **168** | ∞ | ∞ | ∞ |
| **316L** | **Fe** | **168** | 0.55 | 6.6 | N/A |
|  | **Cr** | **168** | 0.0045 | 0.022 | N/A |
|  | **Ni** | **168** | ∞ | ∞ | N/A |
|  | **Mn** | **168** | ∞ | ∞ | N/A |

*<LOD – below limit of detection; N/A – no surface oxide data available;* ∞ - no surface oxide content detected.

*Table S3. Comparison between observed and reported metal release rates for stainless steel grades 304 and 316L in dependence of solution pH*

| **Investigated material, ref** | **Released metal** | **pH** | **Release rate / µg/cm^2^/h** | **Comparison to release rates of this study** |
| --- | --- | --- | --- | --- |
| 304 (abraded as in this study), artificial rain^a^, 4 h of exposure [1] | Ni | 5.7 | 0.0002 | 0.0005 (PBS),  0.006 (PBS+BSA), all 304, 4 h, pH 7.4 |
|  |  | 4.3 | 0.015 |  |
|  | Cr | 5.7 | - | 0.0002(PBS),  0.002 (PBS+BSA), all 304, 4 h, pH 7.4 |
|  |  | 4.3 | 0.005 |  |
| 316L <45µm^b^ powder, as-received^c^, ultra-pure and pH adjusted water, 24 h of exposure [2] | Fe | 6.0 | 0.00015±0.00003 | 0.006 (PBS),  0.06 (PBS+BSA), all 316L, 24 h, pH 7.4 |
|  |  | 4.5 | 0.00009±0.00001 |  |
|  | Cr | 6.0 | 0.00006±0.00001 | 0.00002 (PBS), 0.0005 (PBS+BSA), all 316L, 24 h, pH 7.4 |
|  |  | 4.5 | 0.00025±0.00005 |  |
|  | Ni | 6.0 | 0.0001±0.00003 | 0.0004 (PBS),  0.005 (PBS+BSA), all 316L, 24 h, pH 7.4 |
|  |  | 4.5 | 0.0002±0.00004 |  |
|  | Mn | 6.0 | 0.0025±0.00007 | 0.0003 (PBS),  0.0008 (PBS+BSA), all 316L, 24 h, pH 7.4 |
|  |  | 4.5 | 0.008±0.001 |  |

^a^ Note that this study is based on artificial rain events, not directly comparable to bulk solution exposures.

^b^ Those particles (sized <45µm) are most comparable to massive 316L, as they receive a similar microstructure [3].

^c^ as-received (non-polished) particles are not directly comparable to abraded materials, as abraded materials are expected to release significantly more metals [4].

*Table S4. Literature data on Cr/(Cr+Fe) mass ratio in the outermost surface oxide, investigated by XPS, in dependence of solution and solution pH, and compared to data of this study*

| **Investigated material, ref** | **Solution and pH** | **Cr/(Cr+Fe) / rel. mass %** | **Comparisonwith this study** |
| --- | --- | --- | --- |
| 304 and 316L, and 430 (abraded as in this study), 8 h of exposure [4] | Artificial rain, pH 4.4 | About 0.45 | 0.40-0.45 (304, both BSA and LSZ), after 8 h |
| 304, 316L, and 430 (abraded as in this study), 168 h of exposure [4] | Artificial lysosomal fluid, pH 4.5 | About 0.5 | 0.40 (304, BSA), 0.50 (304, LSZ), 0.65 (316L, BSA), 0.60 (430, BSA), all after 168 h |

*
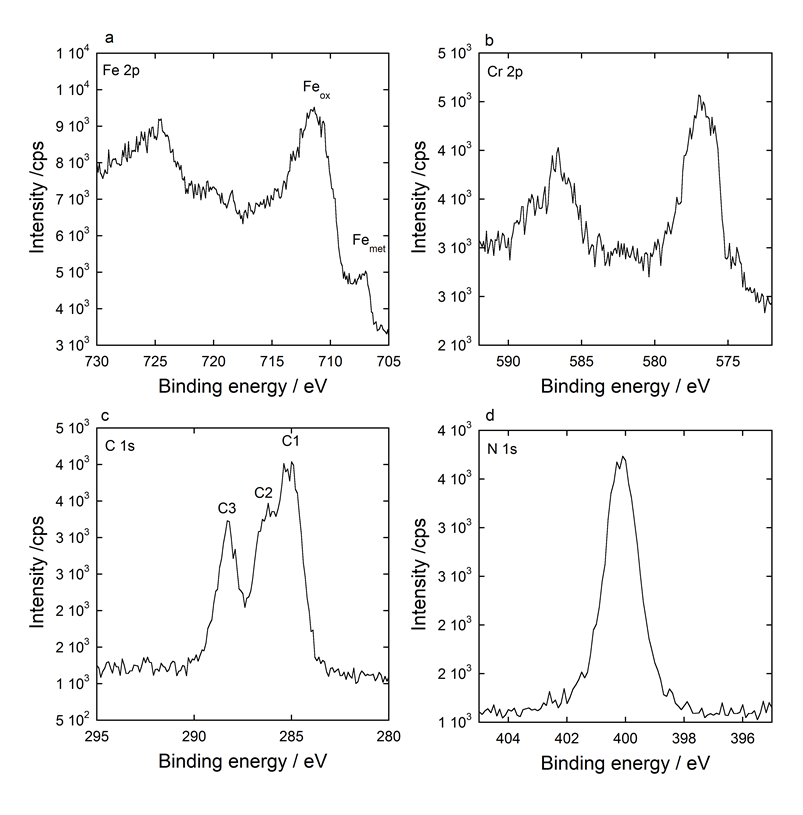
*

*Figure S1. Representative high resolution spectra of Fe 2p (a), Cr 2p (b), C 1s (c), and N 1s (d) monitored by means of X-ray Photoelectron Spectroscopy*

**References**

[1] I Odnevall Wallinder, JS Lu, S Bertling, C Leygraf (2002) Corrosion Science 44: 2303.

[2] Y Hedberg, J Hedberg, Y Liu, I Odnevall Wallinder (2011) BioMetals 24: 1099.

[3] Y Hedberg, O Karlsson, P Szakalos, I Odnevall Wallinder (2011) Materials Letters 65: 2089.

[4] G Herting, I Odnevall Wallinder, C Leygraf (2005) Journal of The Electrochemical Society 152: B23.
